# Potential of Environmental DNA to Evaluate Northern Pike (*Esox lucius*) Eradication Efforts: An Experimental Test and Case Study

**DOI:** 10.1371/journal.pone.0162277

**Published:** 2016-09-14

**Authors:** Kristine J. Dunker, Adam J. Sepulveda, Robert L. Massengill, Jeffrey B. Olsen, Ora L. Russ, John K. Wenburg, Anton Antonovich

**Affiliations:** 1 Alaska Department of Fish and Game, Sport Fish Division, Anchorage, Alaska, United States of America; 2 U.S. Geological Survey, Northern Rocky Mountain Science Center, Bozeman, Montana, United States of America; 3 Alaska Department of Fish and Game, Sport Fish Division, Soldotna, Alaska, United States of America; 4 Conservation Genetics Laboratory, U.S. Fish and Wildlife Service, Anchorage, Alaska, United States of America; University of Hyogo, JAPAN

## Abstract

Determining the success of invasive species eradication efforts is challenging because populations at very low abundance are difficult to detect. Environmental DNA (eDNA) sampling has recently emerged as a powerful tool for detecting rare aquatic animals; however, detectable fragments of DNA can persist over time despite absence of the targeted taxa and can therefore complicate eDNA sampling after an eradication event. This complication is a large concern for fish eradication efforts in lakes since killed fish can sink to the bottom and slowly decay. DNA released from these carcasses may remain detectable for long periods. Here, we evaluated the efficacy of eDNA sampling to detect invasive Northern pike (*Esox lucius*) following piscicide eradication efforts in southcentral Alaskan lakes. We used field observations and experiments to test the sensitivity of our Northern pike eDNA assay and to evaluate the persistence of detectable DNA emitted from Northern pike carcasses. We then used eDNA sampling and traditional sampling (i.e., gillnets) to test for presence of Northern pike in four lakes subjected to a piscicide-treatment designed to eradicate this species. We found that our assay could detect an abundant, free-roaming population of Northern pike and could also detect low-densities of Northern pike held in cages. For these caged Northern pike, probability of detection decreased with distance from the cage. We then stocked three lakes with Northern pike carcasses and collected eDNA samples 7, 35 and 70 days post-stocking. We detected DNA at 7 and 35 days, but not at 70 days. Finally, we collected eDNA samples ~ 230 days after four lakes were subjected to piscicide-treatments and detected Northern pike DNA in 3 of 179 samples, with a single detection at each of three lakes, though we did not catch any Northern pike in gillnets. Taken together, we found that eDNA can help to inform eradication efforts if used in conjunction with multiple lines of inquiry and sampling is delayed long enough to allow full degradation of DNA in the water.

## Introduction

Eradicating invasive species is desirable because their environmental and economic impacts are costly and often irreversible [1]. Successful eradication requires removal of all reproducing individuals, but managers are challenged with evaluating success once the number of remaining individuals falls below detection levels [2]. Populations at very low abundance can be exceedingly difficult to detect using traditional techniques and the unknown survival of only a few individuals can compromise the success of expensive eradication campaigns and result in additional environmental and economic costs [3, 4]. Despite these difficulties, more than 1000 successful invasive species eradications have succeeded worldwide, and targeted surveys for survivors have been a hallmark of many of these successes [5, 6]. Techniques that improve the ability to detect these survivors will help increase the number of successful eradications and minimize invasive species reestablishments that go undetected after eradication efforts.

Non-native fish are a common target of eradication efforts since hundreds of species have been introduced around the word for human consumption and recreation [7]. Non-native fish often outcompete, prey on, or hybridize with native species, causing them to become invasive species in their regions of introduction (e.g., [8, 9, 10]). Consequently, removal of invasive fish is an important need for conservation and restoration of native species in many regions [11]. Even though invasive fish are a pervasive conservation and economic issue across the world, eradication options are largely limited to piscicides. Alternative means of fish eradications, such as dewatering or physical removal with nets, are only viable options under limited conditions [12]. Rotenone is the primary piscicide used in eradication efforts. It affects gill-breathing animals by inhibiting their use of oxygen at the cellular level [13]. Although a number of piscicide applications have achieved complete eradication, many have failed owing to a wide range of factors including the physical water conditions, presence of dense aquatic vegetation that provides refuge, and application methods used (reviewed by [14]). Because of these potential complications and the challenges with detecting target species at low abundances, managers occasionally treat targeted waters with multiple applications of piscicides to ensure eradication. However, each treatment comes at a considerable cost as rotenone applications are expensive, can have non-target effects, and are often socially controversial. Therefore tools that provide timely knowledge about rare survivors of eradication efforts would be useful for continued management in these waters.

Environmental DNA (eDNA) sampling is a relatively new approach that is effective at detecting the presence of low-density aquatic species, including invasive species, but investigations into the application of eDNA for monitoring eradication efforts are only just beginning [15]. The method uses DNA-based identification to detect species from extracellular DNA, or cell debris, that organisms leave behind in the environment [16]. Multiple studies now show that eDNA sampling can be more sensitive at detecting a low-density target species than traditional sampling methods and is less labor intensive (e.g., [17]). However, the potential for false-positives and false-negatives identified in previous studies underscores the need for rigorous testing prior to broad application (e.g., [18]).

Application of eDNA to monitor for survivors of eradication efforts is an obvious extension of this tool; however, the nuances associated with piscicide eradication efforts may limit the reliability of eDNA. In cool lakes, most piscicide-killed fish sink to the bottom and slowly decay [19]. Once extracellular DNA is released from the fish carcasses, it may persist in the environment and provide eDNA evidence of a fish survivor even though all fish are dead. DNA persistence is affected by multiple factors, including endogenous nucleases, water conditions, UV radiation, bacteria and fungi, and these factors operate at a slower rate in cold or dark environments [20]. Because lake bottoms are often cold and have minimal light and piscicide applications in temperate lakes often occur during cooler temperatures (i.e., the fall just prior to freezing) to maximize rotenone toxicity persistence [21], DNA released from fish carcasses under these conditions may persist for long periods and remain detectable with eDNA techniques. It is important to reconcile how carcasses and DNA persistence may complicate the interpretation of positive eDNA detections before eDNA techniques are broadly applied to assess eradication efforts.

We evaluated the efficacy of eDNA sampling to detect invasive Northern pike (*Esox lucius;* hereafter pike) following fall rotenone eradication efforts in southcentral Alaska. Alaska has both native and introduced populations of pike. Its native distribution extends north and west of the Alaska Mountain Range. Immediately south of this region, pike were introduced beginning in the late 1950’s and are today considered an invasive species. Pike are generalist predators that have been introduced into freshwater systems across the globe and have been linked to the decline and elimination of multiple fish species (e.g., [22, 23]). In southcentral Alaska, heightened concern exists over the depletion of wild salmonid (*Oncorhynchus* spp.) populations due to invasive pike predation [8, 24]. Consequently, Alaska natural resource agencies have taken actions to control or eradicate select invasive pike populations (e.g., [25, 26]). In Alaska and other regions where this species is a management concern, eDNA sampling would be a useful tool to evaluate the success of eradication efforts. However, the persistence time of detectable DNA from pike carcasses in southcentral Alaska lakes is likely to be extreme since rotenone applications tend to occur in the fall and lakes in the region are ice-covered and dark throughout the winter and spring.

We used a step-wise field approach to test the efficacy of eDNA as a detection tool for pike presence after eradication efforts. First, we used field observations and experiments to test if our eDNA assay could detect a large free-roaming pike population and low-density caged pike populations. Second, we evaluated how sampling distance from caged pike affected eDNA detection in order to inform field sampling strategies. Third, we assessed the persistence of pike DNA in lakes following pike carcass stocking. Lastly, we compared detection results of gillnetting surveys to eDNA sampling for four connected lakes subjected to a rotenone-treatment designed to eradicate invasive pike. Through this work, we found that eDNA analysis can be an effective tool to test for survivors of eradication efforts if used in conjunction with multiple lines of inquiry and sampling is delayed long enough to allow full degradation of DNA in the water. The success of this project also indicates the potential for eDNA to assist in monitoring the success of other aquatic invasive species eradication efforts.

## Materials and Methods

### Free-roaming pike tests

**Fig 1:**
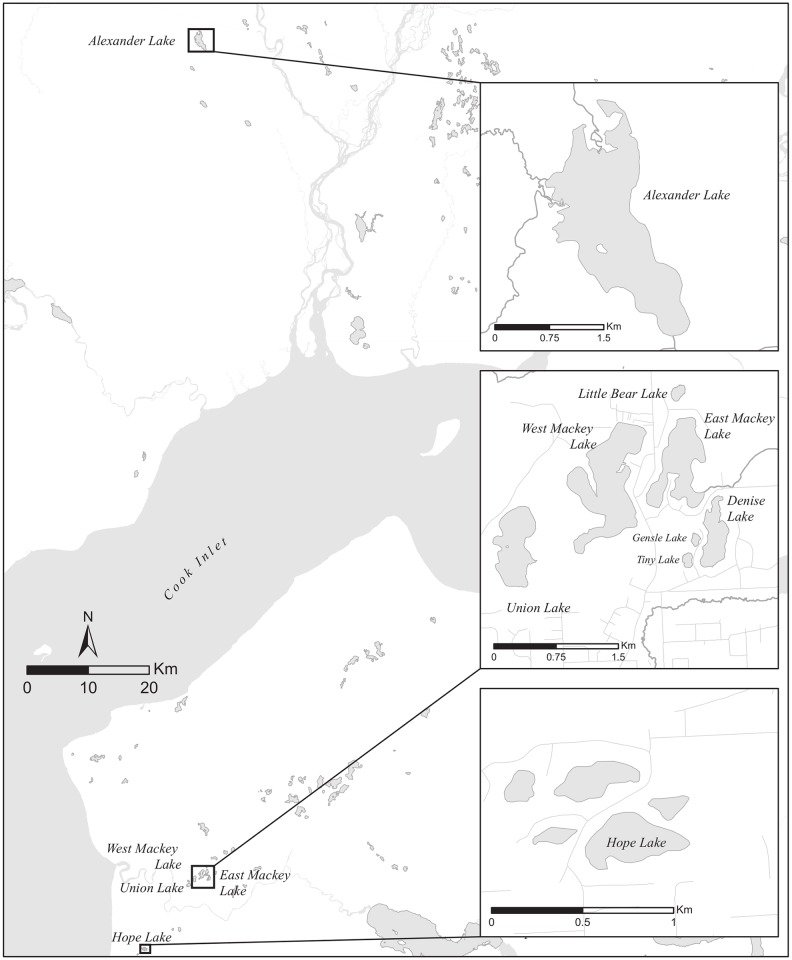
Location of sampled lakes in southcentral Alaska. Alexander Lake is in the Susitna River drainage north of Cook Inlet, while the other lakes occur near Soldotna, AK on the Kenai Peninsula. The background base map is exclusive property of Environmental Systems Research Institute, Inc. (Esri).

We collected water samples from Alexander Lake to assess the efficacy of eDNA detection methods for an unmanipulated invasive pike population. Alexander Lake is located in the headwaters of the Alexander Creek drainage in the Susitna River basin (Fig 1) and has a large invasive pike population that has been present for decades. In 1995, 12,959 (36 spawners per hectare) pike were estimated to be of spawning size (> 300 mm fork length) in Alexander Lake, indicating that a robust population is established [27]. We collected 10, 1-L subsurface samples by submerging sterilized sampling bottles (~ 10 cm) until full. Samples were collected on 8 August 2013 in areas of prime pike habitat (i.e., expansive beds of aquatic macrophytes; [28].

### Caged experiments

**Fig 2:**
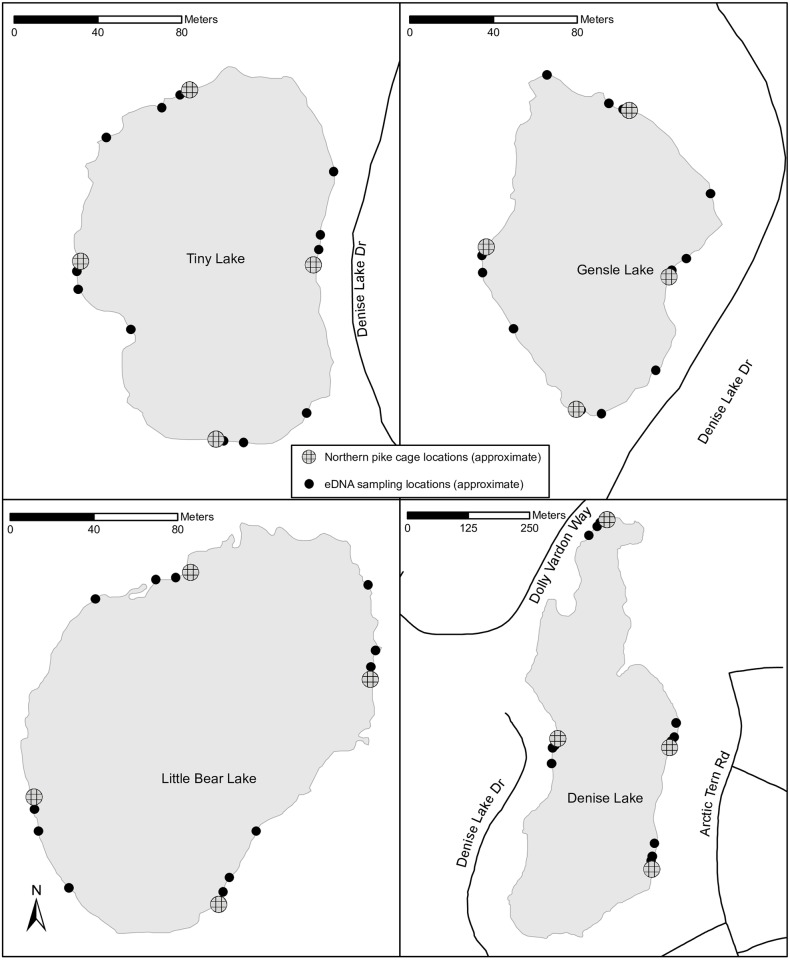
Schematic of the locations of Northern pike cages relative to eDNA sampling locations. Northern pike carcass locations were identical to cage locations in Tiny, Gensle, Little Bear, and Denise lakes near Soldotna, AK. The background base map is exclusive property of Environmental Systems Research Institute, Inc. (Esri).

We then conducted eDNA field tests of low density caged pike in four lakes (Denise, Gensle, Little Bear and Tiny) within the Soldotna Creek drainage on Alaska’s Kenai Peninsula (Figs 1 and 2). These lakes are all closed natural waterbodies ranging in volume from 3.72 to 77.39 Ha-m (Table 1) and the only fish species present is threespine stickleback (*Gasterosteus aculeatus*). We considered the smallest three lakes (Gensle, Little Bear and Tiny) as replicates since they had similar size, morphology, and water quality (Table 1 & Table A in S1 File).

**Table 1:** Physical habitat and fish stocking data for caged and carcass experiments.

			Caged experiments			Carcass experiments		
Lake	Volume (Ha-m)	Quadrant	Date	Pike (g, #)	Density (g / Ha-m)	Date	Pike(g)	Density (g / Ha-m)
**Tiny**	5.53	North	6/3	227, 1	184	6/10	7,479	5410
		South		181, 1			7,479	
		East		308, 1			7,479	
		West		299, 1			7,479	
		*total*		1016, 4			29,915	
**Gensle**	3.72	North	6/4	254, 2	239	6/11	6,538	7030
		South		245, 1			6,538	
		East		209, 1			6,538	
		West		181, 1			6,538	
		*total*		889, 5			26,151	
**Little Bear**	8.57	North	6/5	408, 1	229	6/12	15,466	7219
		South		499, 1			15,466	
		East		508, 1			15,466	
		West		544, 1			15,466	
		*total*		1959, 4			61,863	
**Denise**	77.39	North	6/5	390, 1	18			
		South		327, 2				
		East		345, 1				
		West		336, 1				
		*total*		1398, 5				

Description of lake volume and date (month/day), mass (g), abundance (#) and density (g/ Ha-m) of live (caged experiments) and dead (carcass experiments) pike stocked in four quadrants in four lakes in the Soldotna Creek drainage on Alaska’s Kenai Peninsula in 2013.

In May 2013, we used gill nets (2.54-cm bar mesh) to capture adult pike from Hope Lake near Soldotna, AK (Fig 1). Pike > 300 mm (total length, TL) were captured by their teeth or entangled, while pike < 300 mm TL were often gilled. To minimize pike injury and mortality, we checked gillnets hourly and removed any captured fish. We held fish without obvious external injuries in aerated live boxes in the lake and identified male pike by gamete release, as pike were in spawning condition. We then transported only confirmed male pike to Denise, Gensle, Little Bear and Tiny lakes. Only confirmed male pike were used since we needed to be certain we were not introducing both sexes to the study lakes in case an escape occurred. We placed live pike into cages, which consisted of 208-L polyethylene drums (Mfg. # 1656, Eagle Manufacturing Company; Wellsburg, WV) that were perforated liberally with 1-cm drill holes to permit water exchange. Cages were tethered to metal fence posts. We fully submerged four cages in each lake, with a single cage located along the north, south, east and west axis points near the shoreline (Fig 2). We spaced each cage approximately equidistant from neighboring cages with respect to shoreline perimeter. Pike were held in cages for seven–eight days and did not have access to food. This brief period without food was unlikely to stress pike since this species is well adapted for periods of prolonged starvation [29]. Permission (Fish Transport Permit 13A-0038) for pike capture and caged experiments was given by Alaska Department of Fish and Game (ADFG), the state permitting authority.

**Fig 3:**
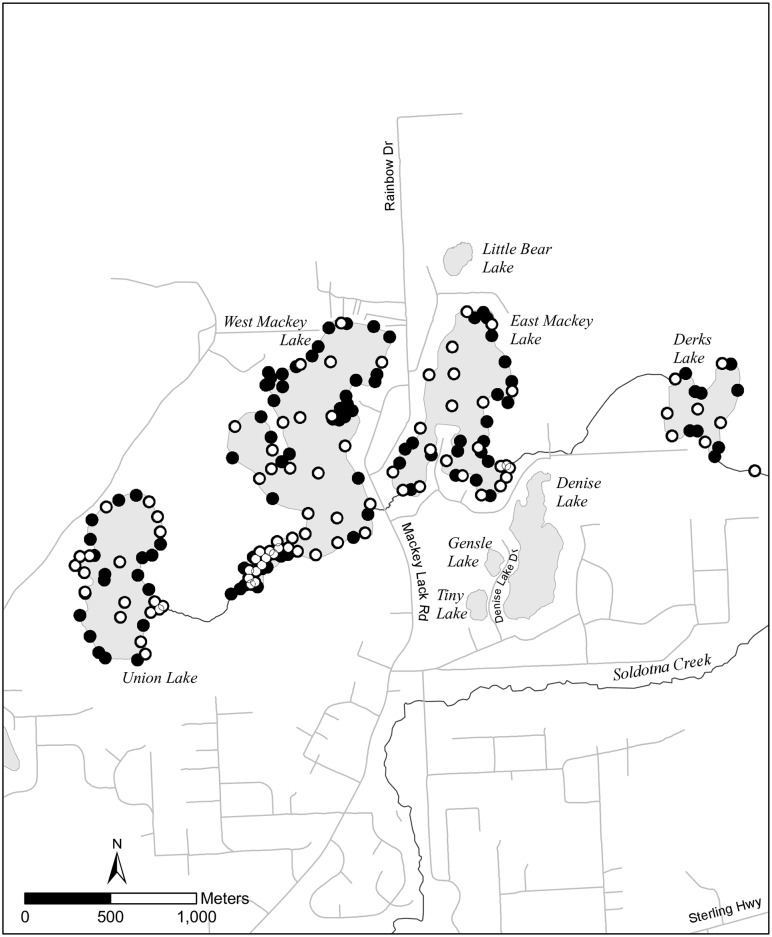
Schematic of the locations of eDNA sampling. Locations of pre (○)and post (●)-rotenone treatments are identified in Derks, East Mackey, Union, and West Mackey Lakes near Soldotna, AK. Post-treatment samples were collected at all pre-treatment sample locations. The background base map is exclusive property of Environmental Systems Research Institute, Inc. (Esri).

At each lake, we stocked cages by weight as evenly as possible beginning on 3 June 2013 (Table 1). In all but two instances, cages were stocked with a single live pike; two cages received two pike each in order to attain a weight similar to other cages in the lake. The three replicate lakes were stocked with similar pike densities (Table 1). The largest lake, Denise Lake, was stocked an order of magnitude less (Table 1). We intended for the range in stocking densities to mimic very low pike densities, similar to what might exist if a small number of fish survived an eradication attempt. For comparison, the density of a long-established invasive pike population in a lake (Derks Lake; Fig 3) within the same drainage was estimated at a minimum of 27,867 grams of pike/ha-m [30].

One week prior to stocking, we collected duplicate 1-L water samples at each lake at four nearshore sites representing each lake’s north, south, east and west axis points. One week after stocking, we collected duplicate 1-L water samples at three distances away from each cage. Sampling sites were within 2 m of the shoreline and located at 1 m, 10 m and 40 m from each cage (Fig 2). Water depths of these sampling sites ranged from 0.5–3.0 m. Forty meters was the maximum distance sampled because exceeding this distance in some of the smaller lakes would have located the sample < 40 m from a neighboring cage.

We used a Nasco swing sampler (Nasco; Fort Atkinson, WI) to collect water in 1-L, sterilized bottles. Each sample was a composite and represented an equal amount of water collected from near the lake bottom, mid-water column and just below the lake surface. At each sampling depth, the collector used the swing sampler to fill a 1-L sterilized bottle and then poured 1/3^rd^ of its contents into the composite bottle. We collected samples in a sequential pattern around the periphery of each lake.

Following the collection of eDNA samples, we collected water quality data using a Quanta Hydrolab (OTT Hydromet; Kempten, Germany) from the middle of each lake near its deepest location (Table A in S1 File). Data records were collected from just above the lake bottom to the lake surface in 1-m increments. Water visibility was measured to the nearest 0.1 m using a Secchi disc. We then removed pike from cages and euthanized them with an overdose of anesthetic (Tricaine methanesulfonate; MS-222), per Alaska Department of Fish & Game (ADFG) regulations.

### Carcass experiments

To evaluate the persistence of pike DNA resulting from carcasses stocked in lakes, we conducted experiments in the three smaller, replicate lakes (Gensle, Little Bear and Tiny) previously used for the live experiments. At each lake, we stocked carcasses to achieve a stocking density between 5,410 to 7,219 grams/Ha-m (Table 1), a range we believed represented a moderately low density population relative to introduced pike densities in regional lakes (e.g., 27,867 grams of pike/ha-m in Derks Lake).

Carcass stockings at each lake were on separate but sequential days beginning on 10 June 2013 (Table 1). We used the same cages and sampling sites used during the live pike trails (Fig 2). It was not necessary to ensure that the lakes were completely free of pike DNA prior to the carcass experiments because our intent was to document DNA persistence under conditions mimicking those following an actual eradication attempt. Cages were stocked with frozen whole or sectioned pike, and at each lake, carcass weights were distributed evenly between cages. We temporarily stocked carcasses in cages to prevent animal savaging until decomposition was advanced and the carcasses were no longer intact and salvageable; this occurred between 19–22 July 2013. We then removed all carcasses from cages and dumped remains into the lake at the exact location the cages occupied in order to expose the remains to more natural and unprotected decomposition processes and minimize potential cage effects.

We collected water samples at days 7 (17–19 June), 35 (15–17 July 1) and 70 (19–21 August) following the stocking of pike carcasses. Duplicate 1-L, composite samples were collected < 1 m from each of the four carcass stocking locations in each lake. Following the collection of eDNA samples at each study lake, we collected water quality data using the same protocols as those previously described (Table A in S1 File).

### Pre and post-eradication sampling

Finally, we compared detection results of gillnetting surveys to eDNA sampling in four lakes in the western branch of the Soldotna Creek drainage: Derks Lake, East Mackey Lake, Union Lake, and West Mackey Lake (Fig 3). The ADFG treated these lakes with rotenone during 6–11 October 2014 to remove invasive pike. We collected multiple 1-L composite samples (N = 85) from the four lakes prior to the treatment (22–24 September 2014), at an average sampling intensity of 1 sample per 4.6 surface hectares (Fig 3 and Table B in S1 File). Post-treatment sampling (N = 179) occurred between 14–28 May 2015, following spring ice-out and turnover which occurred the first and second weeks of May, at an average sampling intensity of one sample per 2.3 surface hectares (Fig 3). Lake ice prevented sampling littoral habitats any earlier, but we posited that this 230 day delay after the rotenone treatment would allow DNA from carcasses to degrade to undetectable concentrations in the lakes during a much cooler period than our carcass trials. Pre and post-sample site selections were subjective, and sites were chosen to target littoral macrophyte beds and bog edges because these habitats produced the highest catches of pike during pre-treatment netting.

For pre-treatment gillnet sampling, we set a total of 68 gillnets in the four lakes just prior to freeze-up between 1–8 November 2013 (Table C in S1 File). All gillnets were experimental sinking nets, 36.6 m long, 1.8 m deep, with six panels of mesh (1 each of 1.3 cm, 1.6 cm, 1.9 cm, 2.5 cm, 3.8 cm, and 5.1 cm). We fished the gillnets continuously using under-ice sets until their removal at ice-out between 1–2 May 2014. Post-treatment, we set a total of 20 gillnets in three lakes (Derks Lake, West Mackey Lake and Union Lake) just prior to freeze-up on 24 October 2014 (Table C in S1 File). The gillnets fished continuously until removal at ice-out between 14–19 April 2015. East Mackey Lake was not gillnetted post-treatment with under-ice sets but was netted after ice out between 21–24 May 2015 with 20 gillnets per day (Table C in S1 File).

The duration that a pike carcass remains identifiable in a gillnet was important to interpreting catch results from under-ice gillnetting surveys used to evaluate pike eradication. Rapid degradation of pike captured in gillnets post-treatment could result in the false conclusion of eradication success. We estimated a minimum pike carcass retention time by entangling 12 adult pike carcasses in a gillnet suspended below the ice at Derks Lake on 13 February 2015. The carcasses were all adult pike between 400 and 550 mm in length. The carcass-loaded gillnet was monitored bi-weekly until ice-out to document the minimum carcass retention time, defined as when the first carcass was lost from the net or became too decomposed to visually identify. Because this exercise was not replicated, results must be interpreted with caution.

### eDNA contamination prevention

To minimize contamination risk, we adopted contamination prevention protocols similar to those described by Laramie et al. [31] and Carime et al. [32]. Precautions included sterilization of all sampling equipment using a 50% bleach solution rinse (50% deionized water: 50% household bleach product containing 8.25% sodium hypochlorite), followed by deionized water rinses between all sampling sites. We also used new latex gloves for each sample collected. When possible, we traveled out of the water by foot to collect samples. To access these sample sites, we wore chest waders that were sterilized with a bleach solution rinse prior to sampling each lake. If a boat was needed due to terrain, we avoided driving the boat atop or beyond a sample site until the sample was collected. We used a bleach rinse solution to sterilize the boat hull and allowed it to air dry before entering the water. In addition, we used separate boats for sampling each lake to prevent possible cross contamination.

To test for contamination during sample collection and filtering, we collected a travel, field, and lab blanks [33]. All blanks consisted of collecting a deionized water sample in a sterile 1-L bottle during the various phases of sample handling. A travel blank was prepared prior to departing and was placed in the same container used to transport all samples throughout the day. Deionized water was taken from the lab and used to collect a field blank, where sampling occurred using the same equipment used to collect the eDNA samples. A lab blank was prepared in the same room where eDNA samples were stored and filtered. All water samples including control blanks were immediately sealed individually in Whirl Pak^®^ (Nasco; Fort Atkinson, WI) bags upon collection and stored on ice in a cooler until filtered.

### eDNA sample processing

We filtered all eDNA samples within 24 hours of collection at an ADFG lab in Soldotna. We used a 120V Geotech^™^ peristaltic pump (Geotech Environmental Equipment, Inc; Denver, CO) to draw water from the sample bottle through a silicon tube filter assembly that incorporated an inline round PVC filter holder. Filters were round, 47-mm nitrocellulose mixed ester membrane (Sterlitech Corporation; Kent, WA). Filter pore size varied from 0.45–1.5 μm due to efforts to resolve filter clogging issues (Table 2; e.g., [34]). However, previous studies suggest that even our largest filter pore size was fine enough to detect fish DNA since 1–10 μm is the most common size class of eDNA from disparate fish species (Common Carp *Cyprinus carpio* and Brook Trout *Salvelinus fontinalis*; [34, 35]). The number of filters required to filter each sample varied from one to five depending on how much organic material was in the sample. We handled all filters with sterilized metal tweezers. We placed all filters from each unique water sample into a single sterile 50-ml centrifuge tube that was then sealed in a Whirl Pak bag, and placed into -20°C storage immediately after filtration.

**Table 2:** Percent frequency of filter pore sizes used to capture eDNA.

	Filter pore size (μm)				
Experiment	0.45	1.0	1.2	1.5	Total (n)
Free-roaming pike			100%		12
Caged, pre	75%			25%	32
Caged, post	21%		79%		105
Carcass			100%		103
Eradication, pre		100%			97
Eradication, post		100%			206

The percent frequency of filter pore sizes used to capture eDNA relative to the total (n) number of water samples, including blanks, collected in each Northern pike eDNA experiment.

After each sample was filtered, we sterilized the tweezers and filter assembly in a 50% bleach solution bath for 10–15 minutes followed by two deionized water baths. Before filtering a new sample, we sprayed the pump and associated work area with a 10% bleach solution and wiped dry. The filter assembly was reassembled and we pumped 0.5–1.0 L of deionized water through as a final rinse. New latex gloves were worn whenever a new sample was handled.

### Genetic methods

We tested all filter samples for pike DNA at the U.S. Fish & Wildlife’s Conservation Genetics Laboratory (Anchorage, AK). DNA was extracted from filter samples using The Qiagen DNeasy^®^ Blood & Tissue Kit and Investigator^®^ Lyse & Spin Kits (Qiagen GmbH, Hilden, Germany). The standard DNeasy protocol was modified to utilize the Lyse&Spin tubes for the filter digest stage. The entire filter was digested and adjusted in 370 μL of ATL buffer and 25 μL of proteinase K for a final volume of 395 μL per sample. A total of 400 μL each of AL and ethanol was added to the supernatant following digestion and discarding of field filters. We combined samples with multiple filters after digestion into a single DNeasy filter. The volumes for Buffers AW1 and AW2 adhered to the DNeasy handbook. The final elution was adjusted to 120 μL of Buffer AE at 55°C. We extracted samples within lakes in small batches (N = 20–30), with three negative controls per extraction batch. Samples from each lake were processed separately to avoid cross contamination. Contamination prevention included UV sterilization of tools and reagents, changing gloves between all samples and sterilizing work surfaces with DNA AWAY^™^. All extractions were done in a room reserved for extracting eDNA samples, where no PCR products or other sources of high concentration DNA are handled.

A previously developed and tested pike specific COI assay was selected to conduct this study: EluCOI F-primer: 5’-CCTTCCCCCGCATAAATAATATAA-3’ R-primer: 5’- GTACCAGCACCAGCTTCAACAC-3’ (Thermo Fischer Scientific Inc., Waltham, MA) and: probe-6FAM-CTTCTGACTTCTCCCC-MBG-NFQ [36, 37]. The assay was conducted using a QuantStudio 12K Flex Real-Time PCR system and 20 μl volume PCR consisting of 10 μl of TaqMan Environmental Master Mix 2.0 (Thermo Scientific Inc.), 2.0 μl of Exogenous Internal Positive Control Reagents (Exo-IPC), 0.4 μl of 50x Exo-IPC DNA, 1 μl COI (20x) assay (primers at 18 μM, probe at 5 μM), and 4 μl of DNA template. The Exo-IPC was used to identify negative results due to absence of the target sequence versus negative results due to PCR inhibition. Cycling conditions were as follows: 50°C for 2 min, 95°C for 10 min followed by 50 cycles of 95°C for 15 s and 60°C for one min. Twelve non-template controls (NTC, 4 μl diH_2_O in place of template) were included on each 96-well assay plate (one per row).

Samples were run in triplicate during qPCR for optimal detection of low concentration or degraded DNA (e.g., [38]). Samples with three positive results were scored positive. For samples with only one or two positive results in the triplicate, we reanalyzed the original sample in triplicate. If any of the wells amplified during a 2^nd^ round, we considered the sample positive. It should be noted that the re-run analysis step initially included 12 replicates for 53 of the live trial samples, including blanks, but was reduced to triplicate for all samples following review of similar studies and efforts to improve cost efficiency (e.g., [38]).

To provide post-hoc insight about unexpected pike eDNA detections in our field experiments, we used quantification cycle (C_q_) values to provide initial insight on the relative DNA copy number of each sample. C_q_ values increase with a decreasing amount of target DNA so C_q_ can provide a relative measure of the copy numbers of target DNA in the PCR reactions [39]. Since PCRs were run for 50 cycles, we used the equation 50 –C_q_ to describe relative DNA copy number. For any replicate that did not amplify, we set C_q_ to 50. This equation assumes reaction efficiency was consistent within and across plates. A standard curve can be used to test this assumption and to estimate absolute quantity of DNA, but standard curves were not run for our analyses because our original objective was to only score samples as positive or negative. Consequently, our C_q_ values provide only initial insight on relative DNA copy number.

### Data analysis

We used generalized linear mixed models (GLMM) to determine the effect of distance on the eDNA detection probability, while treating lakes and quadrants nested within lakes as random effects. Quadrants nested within lakes did not have any effect on eDNA detection so we dropped this nested term from the model. Since we classified each sample as having a “positive” or “negative” eDNA detection at distance *i*, our data are binomial with the probability of success defined as the probability of eDNA detection. We denoted this probability at distance *i* and lake *j* as *π*_*ij*_, and used the logit link function in the GLMM model as:

$$\text{log}[\pi_{ij}/\left(1-\pi_{ij})\right]=\mu +\tau_{i}+c_{j}+\varepsilon_{ij},$$

where *μ* is the intercept, *τ*_*i*_ is the *i*-th distance effect, *c*_*j*_ is the *j*-th lake effect, and *ε*_*ij*_ is the error term. The denominator degrees of freedom for tests of fixed effects were computed by the Satterthwaite method [40]. The model solution was estimated by the iterative residual pseudo-likelihood method and it was run until convergence was reached [40]. To further confirm our decision to drop quadrant from the GLMM, we also used chi-square tests of contingency tables to compare eDNA detection rates among geographical quadrants within and among lakes. All modeling was done using SAS/STAT software, Version 9.3 of the SAS System for Windows.

We used descriptive statistics (e.g., range and mean) to assess the efficacy of eDNA in Alexander Lake, the persistence of pike DNA resulting from carcasses stocked in lakes, and to compare pre and post-treatment eDNA and gill net sampling in rotenone-treated lakes. Differences among these treatments were large and did not require formal statistics. Finally, we used post-hoc estimates of relative DNA copy number (50 –C_q_) to provide insight about unexpected positive detections. Because C_q_ assumptions could not be tested, we only qualitatively compared these estimates.

## Results

### Free-roaming pike tests

We detected pike DNA in 9 of 10 samples (90%) collected from Alexander Lake. For eight of these positive detections, all replicates were positive (i.e., 3/3). For the ninth positive detection, two of three replicates were positive on the first run and 12 of 12 replicates were positive on the second run. All three replicates for the negative sample were negative and all control blanks were negative. All Exo-IPC assays were positive with the expected qPCR curves indicating that there was no inhibition for the samples in this test and throughout the study.

### Caged experiments

**Fig 4:**
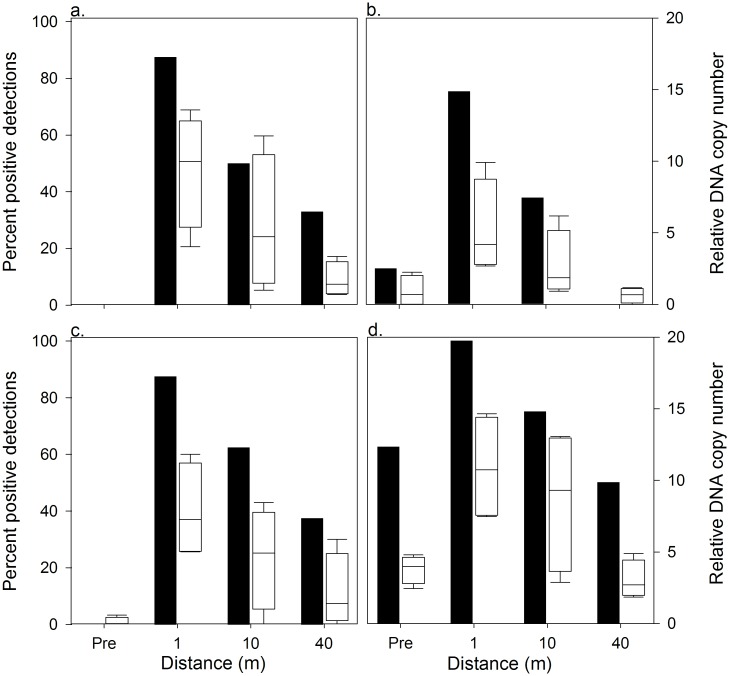
Percent positive detections and relative DNA copy number for Northern pike cage experiments. Percent positive detections (filled bars) and relative DNA copy number (unfilled, box plots) for Northern pike cage experiments in Denise (a), Gensle (b), Little Bear (c) and Tiny (d) Lakes near Soldotna, AK. For the box plots, the dark horizontal line represents the mean, with the box representing the 25^th^ and 75^th^ percentiles and the whiskers the 5^th^ and 95^th^ percentiles. Water samples (n = 8 per lake treatment) were analyzed for Northern pike DNA prior (Pre) to introduction of caged Northern pike and then 7 days after introductions at 1 m, 10 m, and 40 m away from each cage.

Prior to stocking caged pike, we unexpectedly detected pike DNA in Gensle Lake (1 of 8 samples; 12.5%) and Tiny Lake (5 of 8 samples; 62.5%; Fig 4). We detected pike DNA in one triplicate subsample in Little Bear Lake, but we scored this sample as negative because no DNA amplified in a second run. Pike DNA was not detected in Denise Lake prior to stocking (Fig 4 and Table B in S1 File). Seven days after stocking, we detected pike DNA in all four lakes (Fig 4 and Table B in S1 File). Detections occurred at all sample distances in all lakes, with the exception that there were no detections at 40 m in Gensle Lake. There was no effect of quadrant on detection rate (χ^2^ = 0.13, df = 3, *P* = 0.06) and, in the GLMM, the random effects of lake and quadrant nested within lake explained 14% and ~ 0% of the variance, respectively. The distance effect on the detection rate was highly significant (GLMM: F = 9.90, *P* < 0.01). Probability (SE) of DNA detection was estimated to be 88.6% (6.7%) at 1 m, 56.6% (12.9%) at 10 m, and 26.9% (10.8%) at 40 m. In terms of odds ratios (95% C.I.), we were 21(5.3–85.1) times more likely to detect DNA at 1 m than at 40 m. The odds of detecting DNA were 6 (1.6–22.6) times higher at 1 m than at 10 m. All control blanks were negative. Relative DNA copy numbers for the positive detections prior to stocking caged pike were similar to copy numbers for 40 m water samples. However, relative DNA copy numbers from both of these sampling periods were smaller than copy numbers for 1 m and 10 m water samples (Fig 4). The range of relative DNA copy numbers decreased with distance from caged pike.

### Carcass Experiments

**Fig 5:**
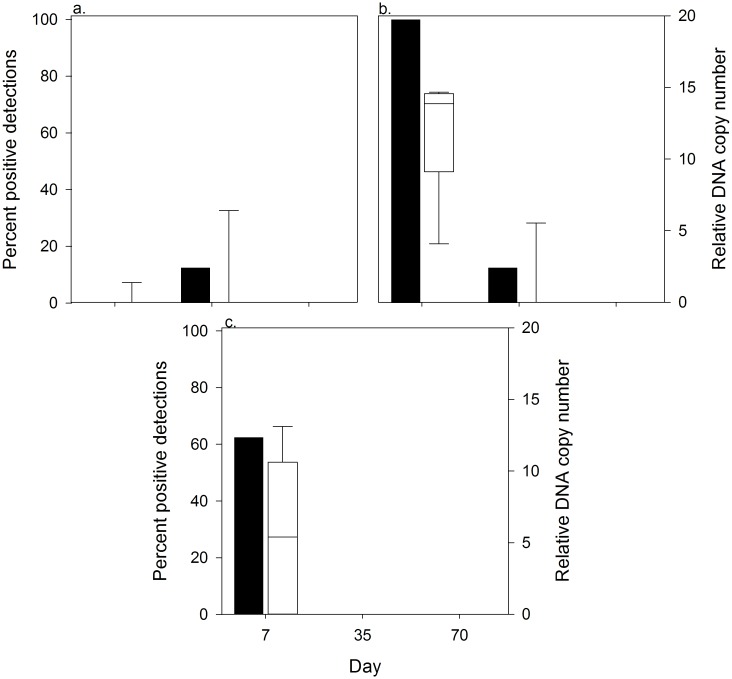
Percent positive detections and relative DNA copy number for Northern pike carcass experiments. Percent positive detections (filled bars) and relative DNA copy number (unfilled, box plots) for Northern pike carcass experiments in Gensle (a), Little Bear (b) and Tiny (c) Lakes near Soldotna, AK. For the box plots, the dark horizontal line represents the mean, with the box representing the 25^th^ and 75^th^ percentiles and the whiskers the 5^th^ and 95^th^ percentiles. Water samples (n = 8 per lake per day) were analyzed for Northern pike DNA 7, 35 and 70 days after carcass additions.

At day 7 of the carcass experiments, positive detections occurred in Little Bear Lake (8 of 8; 100%) and Tiny Lake (5 of 8; 65.2%), but not in Gensle Lake (Fig 5 and Table B in S1 File). The pooled detection rate was 54.2%. At day 35, we had a single detection of pike DNA at Gensle Lake (1 of 8; 12.5%) and Little Bear Lake (1 of 8; 12.5%). The pooled detection rate was 8.3%. At day 70, we did not detect pike DNA in any lake (Fig 5 and Table B in S1 File). Water temperature ranged between 10.2°C and 20.0°C during the day 7 sampling event, rose to 18.4°C to 20.2°C during the day 35 sampling event, then declined to 10.4°C to 17.0°C during the day 70 sampling event (Table A in S1 File).

We had a single positive lab blank during the carcass trials and it was associated with the day 7 sampling event at Tiny Lake. If we remove day 7 Tiny Lake results from our analysis, we still observe that Little Bear and Gensle lakes each had positive detections at day 7 (Little Bear) and day 35 (Gensle and Little Bear), which indicates that DNA from carcasses can persist in lake water.

Relative DNA copy numbers followed the same spatial and temporal patterns as positive detections rates (Fig 5). At day 7, water samples from Little Bear Lake had more DNA copies than Gensle and Tiny lakes; however, among sample variability in copy numbers from Little Bear Lake was very high. For day 35 and 70, copy numbers were near zero.

### Pre and post-eradication sampling

**Fig 6:**
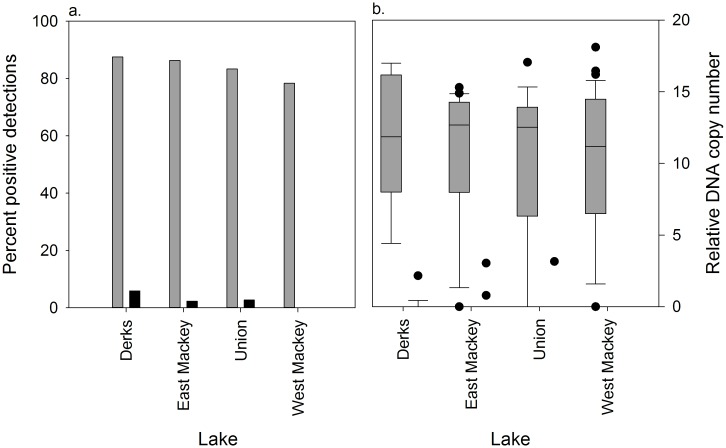
Percent positive detections and relative DNA copy number before and after rotenone eradication treatments. (a) Percent positive detections and (b) relative DNA copy number before (gray filled) and after (black filled) rotenone eradication treatments in Derks, East Mackey, Union and West Mackey lakes near Soldonta, AK. Relative DNA copy numbers are displayed as box plots, with the dark horizontal line representing the mean, the box representing the 25^th^ and 75^th^ percentiles, the whiskers representing the 5^th^ and 95^th^ percentiles and the filled circles representing outliers. eDNA water samples were collected ~ 30 days before and ~ 230 days after the rotenone treatments.

We detected pike DNA in 70 of 85 pre-eradication samples (82.4%; Fig 6 and Table B in S1 File). Post-eradication, we detected pike DNA in 3 of 179 samples (1.7%; Fig 6). Post-eradication detections consisted of a single detection each at Derks (n = 17), East Mackey (n = 44), and Union (n = 37) lakes. One additional triplicate subsample in East Mackey had pike DNA, but it was scored negative since no DNA amplified in the second run. There were no post-treatment detections in West Mackey Lake (n = 81). Relative DNA copy numbers of pre-eradication water samples were large and had high variance at all lakes (Fig 6). The post-eradication DNA copy numbers were near zero (Fig 6). All control blanks were negative.

The gillnetting effort between all four lakes totaled 293,645 hours during the pre-eradication period and 78,336 hours post-eradication (Table C in S1 File). More gillnetting effort was expended during the pre-eradication effort to support ADFG’s goal of removing the invasive pike population. When adjusted for the estimated minimum gillnet carcass retention time (48 days), the pre-eradication gillnetting represented 78,336 hours of gillnetting effort and the post-eradication gillnetting represented 23,472 hours of effort (Table C in S1 File). An estimated 1,825 pike were collected during pre-eradication gillnetting (Table C in S1 File). An actual count of the pike captured was not possible due to the advanced state of decomposition of some carcasses. No pike, or any other species of fish, were captured by gillnets post-eradication.

We inspected net entangled pike carcasses used in the under-ice gillnet retention trial on Derks Lake between 13 February 2015 and 7 April 2015. All carcasses remained entangled and visually identifiable after 48 days of submersion. One carcass was no longer in the net when inspected at day 53. Based on the advanced state of decomposition of the remaining carcasses, we assumed the carcass was lost due to natural degradation processes and was not scavenged. Therefore, we considered 48 days as the minimum time a net entangled pike carcass would be retained in a gillnet when submerged under lake ice. The range in average water column temperature recorded in Derks Lake during the gillnet carcass retention trial was 4.5°C ± 0.8°C.

## Discussion

Eradication of invasive fish with piscicides is a management tool that is used across the world to conserve vulnerable native species. However, managers are challenged with knowing when elimination has been achieved because detecting populations at very low abundances is difficult. For this reason, many have hoped that the application of highly sensitive and specific eDNA techniques can provide an effective and efficient means for detecting survivors. In this study, we found that nonliving sources (e.g., carcasses) and spatial heterogeneity in DNA distribution can complicate eDNA detection results and that multiple eDNA sampling events in conjunction with traditional sampling approaches are required to make strong inferences about eradication success.

Our conclusions are based on results from a stepwise approach that we used to inform where and when to sample for pike DNA in shallow lakes. First, we collected water samples from Alexander Lake to test if our assay could detect a free-roaming, invasive pike population. We subjectively sampled prime pike habitat and had a 90% (i.e., 9 of 10 samples) detection rate. Next, we used live experiments to evaluate how DNA detection is affected by distance from the DNA source (i.e., caged live pike). After seven days, we found that pike DNA slowly diffused away from the source, such that the odds of detecting DNA decreased with distance. Consequently, we targeted eDNA sampling for carcass experiments 1 m from the source and we targeted eDNA sampling in rotenone treated lakes in prime pike habitat. Finally, we stocked pike carcasses into multiple lakes without live pike and found that DNA was detectable for < 70 days in 10–20°C water temperatures. Because water temperatures were < 10°C during our rotenone treatments, we waited > 70 days to sample for pike DNA in four lakes that were treated with rotenone to eradicate pike and ~ 98% of these eDNA water samples were negative. Integration of the post-eradication eDNA results with multiple lines of evidence provided high confidence that no pike survived the eradication efforts.

We found evidence of positive eDNA detections potentially resulting from historical or nonliving pike presence in our live experiments and in our post-eradication sampling. In our live experiments, we unexpectedly found pike DNA in Gensle and Tiny lakes in pre-stocking samples, even though (1) no pike were caught in intensive gillnetting surveys preceding the study, (2) eDNA sampling occurring after 17 June 2013 at Gensle Lake and 16 July 2013 at Tiny Lake failed to detect pike DNA, and (3) the relative DNA copy numbers from these samples were near zero. These results suggest either a temporary or latent DNA source was present (e.g., animal feces, sediment trapped DNA) or the samples were contaminated during handling. Unfortunately, no control blanks were collected during the pre-stocking sample collections that may have helped identify if sample contamination occurred. However, we collected 78 control blanks for our other study components and found only one false positive (1% of controls), suggesting that contamination was a rare occurrence. Regardless of the cause of the pre-stocking detections, the potential bias of these detections on our live trial eDNA detection rates was minor. We eliminated the Gensle and Tiny lakes live trial samples from the pooled detection samples and found no change for the 1-m detection rate (Δ = +0.0%) and relatively minor changes to the 10-m (Δ = +3.9%) and 40-m (Δ = +2.2%) detection rates.

A plausible explanation is that pre-stocking eDNA detections may have resulted from DNA preserved in sediment. Pike were illegally introduced to Tiny Lake at an unknown date and first confirmed in 2010. ADFG attempted to eradicate the Tiny Lake pike population in 2011 through gillnetting efforts, wherein ~ 30 individuals were removed. No further pike were captured despite intensive gillnetting efforts in successive years and immediately prior to this study. We also detected no pike DNA at day 35 and 70 of the carcass experiment, which is additional evidence that an undetected low-density population was not present. Although pike have never been detected at Gensle Lake, it is conceivable that an illegal introduction was attempted but failed to result in an established population, as Gensle Lake lies just 100 m north of Tiny Lake and both lakes are equally accessible by road. Assuming both lakes had pike introductions at some point, their DNA may have been preserved in the sediment. A similar explanation is also plausible for the three positive detections in our post-eradication sampling effort, since our eDNA sampling occurred shortly after lake turnover in the spring. Recent studies indicate DNA can be preserved for many years in the sediment [41, 42]. This latent and potentially irregular source of DNA that is not conditional on current pike presence presents challenges when using highly sensitive eDNA to monitor for post-eradication survivors, as there is a non-zero probability of a positive detection after an eradication effort regardless of success. A better understanding of the factors that influence the release of DNA preserved in the sediment, such as fall and spring mixing events, is required to inform sampling protocols and overcome this challenge. In the interim to these advances, repeat eDNA sampling and multiple lines of evidence may help minimize these complications. We found that multiple eDNA samples collected over time in combination with intensive gillnetting and prolonged, lethal concentrations of rotenone provided strong evidence that the DNA source was not from living pike, which underscores that one-time eDNA sampling, as a sole measure, can provide misleading snapshots of target species presence.

DNA sourced from pike carcasses also results in positive detections. In our carcass experiments, we found that DNA remained detectable at 35 days though the percent of positive detections and relative DNA copy numbers were lower than at seven days (Fig 5). After 70 days, no detectable DNA was found in sampled water. Based on this suggested timeline of DNA carcass persistence, the cooler water temperatures that slow DNA decay [43], and access limitations due to lake ice, we sampled ~230 days post-treatment in four lakes and detected pike DNA in a single water sample at Derks, East Mackey, and Union lakes (1.7% of all post treatment samples). Derks Lake was the site of our gillnet carcass retention trial that lasted until ice-out in the spring of 2014. Most of the pike carcasses used in the trial were deposited in the lake at the conclusion of the trial. In East Mackey and Union lakes, pike carcasses were observed frozen into the lake ice post-treatment and they remained there until ice-out when they presumably dropped to the lake bottom. When these three positive detections are put in the context of (1) the 176 negative detections post-treatment in these four lakes, (2) our post-treatment carcass observations and gillnet results, (3) the much higher percent positive detections and relative DNA copy numbers from our live experiments and pre-treatment surveys, and (4) DNA persistence time results from our carcass experiments, it suggests they resulted from DNA associated with carcasses or sediment, not live fish. These results also underscore that eDNA water sampling must be delayed long enough to allow full degradation of DNA sources from carcasses. A mechanistic understanding of DNA decay is needed to determine the time at which eDNA sampling provides results that are not confounded by piscicide-killed fish.

The distribution of DNA in space can also cause imperfect eDNA detections. We found that the odds of detecting DNA decreased with distance from the DNA source, indicating that pike DNA did not homogenize in the lakes within seven days. In these live experiments, pike were confined to cages so it is unknown how free-roaming pike behavior influences DNA distribution. Our eDNA results from the free-roaming pike population in Alexander Lake show that detection probability is high, but potentially patchy since we did have one negative detection. Previous studies that introduced target taxa into unoccupied streams also found that the DNA signal decays over distance [44, 45]. For free-roaming populations in streams, detection distances were much greater but DNA concentrations were still patchy in space (e.g., [17, 33, 46]). Much less is known about DNA transport in lentic waters, though previous studies do indicate that DNA in lakes is patchy [47, 48]. Our estimates of relative DNA copy numbers from the caged and carcass experiments and from pre-eradication water samples also indicate high within-lake variability, perhaps because our study lakes were relatively small (3.72 to 77.39 Ha-m) and less prone to developing wind generated currents to facilitate mixing. Due to the heterogeneity demonstrated in our study and in past studies, high intensity sampling [49], biologically informed sampling (e.g., eDNA hotspots; [47]), or sampling larger volumes of water [34] may be required to detect surviving target taxa after eradication efforts.

Though our results highlight the limitations of interpreting eDNA data, they also inform managers how to move forward with applying this sensitive sampling tool. As previously mentioned, a repeat eDNA sampling approach can be used to bracket inference strength of positive detections [17]. Multiple positives or increases in DNA concentrations across time would suggest potential of survivors, while the opposite would suggest DNA coming from latent or temporary sources. Because eDNA sampling is rapid, inexpensive and efficient relative to traditional methods [17, 33], sites with positive detections can be targeted with higher intensity eDNA sampling. Managers can also take advantage of the patchy distribution of DNA and the relationship between eDNA probability of detection and distance from DNA source (this study and [47]) to target use of traditional techniques (e.g., gillnets). Regardless of how eDNA is used to inform additional sampling, our study demonstrates that eDNA techniques provide the most information about populations at low abundance when there is temporal replication and they are couched within the context of multiple lines of evidence.

## Supporting Information

S1 File Table A. Water quality data of lakes used in the caged and carcass experiments. Table B. eDNA detection and PCR results for the caged, carcass and rotenone experiments. Table C. Gillnetting data from the pre and post-rotenone treated lakes.
(DOCX)
