# Correction: Potential of Environmental DNA to Evaluate Northern Pike (*Esox lucius*) Eradication Efforts: An Experimental Test and Case Study

**DOI:** 10.1371/journal.pone.0173837

**Published:** 2017-03-08

**Authors:** Kristine J. Dunker, Adam J. Sepulveda, Robert L. Massengill, Jeffrey B. Olsen, Ora L. Russ, John K. Wenburg, Anton Antonovich

There is an error in the third and fourth sentences of the first paragraph under the subheading “Pre and post-eradication sampling” in the Materials and Methods section. The correct sentences are: We collected multiple 1-L composite samples (N = 85) from the four lakes prior to the treatment (22–24 September 2014), at an average sampling intensity of 1 sample per 1.9 surface hectares (Fig 3 and Table B in S1 File). Post-treatment sampling (N = 179) occurred between 14–28 May 2015, following spring ice-out and turnover which occurred the first and second weeks of May, at an average sampling intensity of one sample per 0.9 surface hectares (Fig 3).
