# Supplementary material for: Potential of Environmental DNA to Evaluate Northern Pike (Esox lucius) Eradication Efforts: An Experimental Test and Case Study
Source: PLoS One. 2016 Sep 14;11(9):e0162277. doi: 10.1371/journal.pone.0162277 (PMC5023132; doi:10.1371/journal.pone.0162277)
Supplement: S1 File — Table A. Water quality data of lakes used in the caged and carcass experiments. Table B. eDNA detection and PCR results for the caged, carcass and rotenone experiments. Table C. Gillnetting data from the pre and post-rotenone treated lakes. (DOCX) [file pone.0162277.s001.docx]

S1 Table A

Water quality data from four lakes in the Soldotna Creek drainage on Alaska’s Kenai Peninsula used in the Northern pike caged and carcass experiments. All data were sampled in 2013 and collected from the middle of each lake near its deepest location at 1-m increments throughout the water column, beginning with the surface. We report water temperature (Temp, ° C), specific conductance (SpC, mS/cm), dissolved oxygen (DO, mg/L) and pH as averages (±SD) and water depth (Depth, m) and Secchi disk depth (Secchi, m) as integrated measurements. Secchi disk depths were not available (n/a) for all sampling dates.

| **Lake** | **Date** | **Depth** | **Temp** | **SpC** | **DO** | **pH** | **Secchi** |
| --- | --- | --- | --- | --- | --- | --- | --- |
| Tiny | 6/3 | 3.0 | 12.5 (2.4) | 0.02 (0.00) | 9.6 (0.6) | 7.6 (0.1) | 3.3 |
|  | 6/10 | 3.2 | 16.5 (1.8) | 0.02 (0.00) | 10.0 (1.0) | 8.0 (0.2) | n/a |
|  | 6/17 | 3.5 | 20.0 (1.5) | 0.02 (0.00) | 11.7 (1.9) | 8.0 (0.2) | 3.3 |
|  | 7/16 | 3.5 | 20.2 (0.4) | 0.02 (0.00) | 11.7 (1.9) | 8.0 (0.2) | 2.1 |
|  | 8/19 | 3.5 | 17.0 (0.0) | 0.02 (0.00) | 7.4 (0.3) | 7.1 (0.1) | 1.8 |
|  |  |  |  |  |  |  |  |
| Gensle | 6/4 | 3.5 | 11.0 (4.0) | 0.03 (0.00) | 8.0 (0.7) | 7.3 (0.1) | n/a |
|  | 6/11 | 3.5 | 14.9 (5.6) | 0.03 (0.00) | 8.8 (0.5) | 7.7 (0.3) | 3.0 |
|  | 6/18 | 3.5 | 16.4 (5.7) | 0.03 (0.00) | 10.0 (0.8) | 7.7 (0.3) | 2.3 |
|  | 7/17 | 3.5 | 18.4 (2.0) | 0.03 (0.00) | 7.5 (1.7) | 7.9 (0.4) | 2.1 |
|  | 8/20 | 3.5 | 16.8 (0.2) | 0.03 (0.00) | 7.9 (0.0) | 7.3 (0.9) | 1.3 |
|  |  |  |  |  |  |  |  |
| Little Bear | 6/5 | 6.0 | 7.8 (4.9) | 0.03 (0.01) | 4.4 (4.2) | 6.8 (0.3) | 1.3 |
|  | 6/13 | 6.0 | 10.0 (7.9) | 0.03 (0.01) | 3.7 (3.6) | 7.4 (0.6) | 1.2 |
|  | 6/20 | 6.0 | 10.2 (7.9) | 0.03 (0.01) | 4.5 (4.3) | 7.3 (0.6) | 1.2 |
|  | 7/18 | 6.0 | 18.4 (7.3) | 0.03 (0.02) | 7.5 (3.5) | 7.9 (0.6) | 2.1 |
|  | 8/21 | 6.5 | 10.4 (5.2) | 0.04 (0.02) | 3.4 (4.0) | 6.5 (0.3) | 1.5 |
|  |  |  |  |  |  |  |  |
| Denise | 6/6 | 6.0 | 11.2 (3.7) | 0.02 (0.00) | 9.3 (0.3) | 7.4 (0.2) | 3.5 |
|  | 6/12 | 6.5 | 13.2 (5.4) | 0.02 (0.00) | 9.5 (0.8) | 8.4 (0.5) | 4.0 |

|  | |  |  |  |  | **Positive PCRs** | | | |
| --- | --- | --- | --- | --- | --- | --- | --- | --- | --- |
| **Experiment** | | **Lake** | **Treatment** | **n** | **Positive** | **0/3** | **1/3** | **2/3** | **3/3** |
| Caged | Denise | | Pre | 8 | 0 | 8 | 0 | 0 | 0 |
|  |  |  | 1 m | 8 | 7 | 1 | 1 | 2 | 4 |
|  |  |  | 10 m | 8 | 4 | 3 | 2 | 1 | 2 |
|  |  |  | 40 m | 8 | 2 | 3 | 4 | 1 | 0 |
|  |  | |  |  |  |  |  |  |  |
|  | Gensle | | Pre | 8 | 1 | 6 | 2 | 0 | 0 |
|  |  |  | 1 m | 8 | 6 | 1 | 4 | 1 | 1 |
|  |  |  | 10 m | 8 | 3 | 3 | 4 | 1 | 0 |
|  |  |  | 40 m | 8 | 0 | 7 | 1 | 0 | 0 |
|  |  | |  |  |  |  |  |  |  |
|  | Little Bear | | Pre | 8 | 0 | 7 | 1 | 0 | 0 |
|  |  |  | 1 m | 8 | 7 | 1 | 2 | 3 | 2 |
|  |  |  | 10 m | 8 | 5 | 2 | 3 | 2 | 1 |
|  |  |  | 40 m | 8 | 3 | 4 | 2 | 2 | 0 |
|  |  | |  |  |  |  |  |  |  |
|  | Tiny | | Pre | 8 | 5 | 2 | 3 | 3 | 0 |
|  |  |  | 1 m | 8 | 8 | 0 | 2 | 1 | 5 |
|  |  |  | 10 m | 8 | 6 | 1 | 2 | 2 | 3 |
|  |  |  | 40 m | 8 | 4 | 3 | 4 | 1 | 0 |
|  |  | |  |  |  |  |  |  |  |
| Carcass | Gensle | | 7 d | 8 | 0 | 7 | 1 | 0 | 0 |
|  |  |  | 35 d | 8 | 1 | 7 | 1 | 0 | 0 |
|  |  |  | 70 d | 8 | 0 | 8 | 0 | 0 | 0 |
|  |  | |  |  |  |  |  |  |  |
|  | Little Bear | | 7 d | 8 | 8 | 0 | 1 | 1 | 6 |
|  |  |  | 35 d | 8 | 1 | 7 | 0 | 1 | 0 |
|  |  |  | 70 d | 8 | 0 | 8 | 0 | 0 | 0 |
|  |  | |  |  |  |  |  |  |  |
|  | Tiny | | 7 d | 8 | 5 | 3 | 2 | 1 | 2 |
|  |  |  | 35 d | 8 | 0 | 8 | 0 | 0 | 0 |
|  |  |  | 70 d | 8 | 0 | 8 | 0 | 0 | 0 |
|  |  | |  |  |  |  |  |  |  |
| Rotenone | Derks | | Before | 8 | 7 | 0 | 1 | 3 | 4 |
|  |  |  | After | 17 | 1 | 16 | 0 | 1 | 0 |
|  |  | |  |  |  |  |  |  |  |
|  | East Mackey | | Before | 22 | 19 | 2 | 4 | 1 | 14 |
|  |  |  | After | 44 | 0 | 42 | 2 | 0 | 0 |
|  |  | |  |  |  |  |  |  |  |
|  | Union | | Before | 18 | 15 | 2 | 4 | 1 | 11 |
|  |  |  | After | 37 | 1 | 36 | 0 | 1 | 0 |
|  |  | |  |  |  |  |  |  |  |
|  | West Mackey | | Before | 37 | 32 | 3 | 7 | 9 | 18 |
|  |  |  | After | 81 | 0 | 81 | 0 | 0 | 0 |

S1 Table B

Detection and PCR results of Northern pike eDNA samples (n) collected for the caged, carcass and rotenone experiments. Each water sample was analyzed in triplicate and a sample was considered positive for Northern pike DNA if all three samples were positive (3/3). Samples with one (1/3) or two (2/3) positive results in the triplicate were run a second time in triplicate.

S1 Table C

Pre and post-rotenone treatment gillnetting data from four lakes in the Soldotna Creek drainage on Alaska’s Kenai Peninsula. Gillnets were fished over winter in 2013/2014 prior to rotenone treatment and in 2014/2015 after rotone treatment. We report effort as the total hours (hr) that gillnets were fished. We also adjusted effort for the minimum carcass retention time of 48 days.

| Time | Lake | Set date | Pull date | Gillnets (#) | Effort (hr) | Effort adjusted (hr) | Pike catch (#) |
| --- | --- | --- | --- | --- | --- | --- | --- |
| Pre | Derks | 11/1/2013 | 5/2/2014 | 29 | 126,672 | 33,408 | 650 |
|  | East Mackey | 11/8/2013 | 5/1/2014 | 7 | 29,235 | 8,064 | 600 |
|  | Union | 11/8/2013 | 5/2/2014 | 9 | 37,768 | 10,368 | 300 |
|  | West Mackey | 11/1/2013 | 5/1/2014 | 23 | 99,969 | 26,496 | 275 |
|  | *Total* |  |  | 68 | 293,645 | 78,336 | 1,825 |
|  |  |  |  |  |  |  |  |
| Post | Derks | 10/24/2014 | 4/19/2015 | 6 | 25,472 | 6,912 | 0 |
|  | East Mackey | 4/21/2015 | 4/24/2015 | 20 | 432 | 432 | 0 |
|  | Union | 10/24/2014 | 4/15/2015 | 8 | 33,329 | 9,216 | 0 |
|  | West Mackey | 10/24/2014 | 4/19/2015 | 6 | 25,001 | 6,912 | 0 |
|  | *Total* |  |  | 40 | 84,234 | 23,472 | 0 |
